# News media impact on sociopolitical attitudes

**DOI:** 10.1371/journal.pone.0264031

**Published:** 2022-03-09

**Authors:** Megan Earle, Gordon Hodson

**Affiliations:** Department of Psychology, Brock University, St. Catharines, Ontario, Canada; Carnegie Mellon Univeristy, UNITED STATES

## Abstract

In the present project we assessed whether partisan news affects consumers’ views on polarizing issues. In Study 1 nationally representative cross-sectional data (N = 4249) reveals that right-leaning news consumption is associated with more right-leaning attitudes, and left-leaning news consumption is associated with more left-leaning attitudes. Additional three-wave longitudinal data (N = 484) in Study 2 reveals that right-leaning news is positively (and left-leaning news is negatively) associated with right-leaning issue stances three months later, even after controlling for prior issue stances. In a third (supplemental) study (N = 305), random assignment to right-leaning (but not left-leaning) news (*vs*. control) experimentally fostered more right-leaning stances, regardless of participants’ previously held political ideology. These findings suggest that partisan news, and particularly right-leaning news, can polarize consumers in their sociopolitical positions, sharpen political divides, and shape public policy.

## Introduction

Early in the 20th century news organizations aimed for objectivity by providing both sides of political debates [1,2]. Now, with greater availability and more choices [3,4], many news organizations provide more overt ideological perspectives to differentiate themselves in a more competitive market [5–9]. Many speculate that this partisan news could be driving political polarization [10–12] and some scholars argue that increasing polarization in the United States may alter how people form attitudes [13], thus making them more inclined to uncritically accept arguments put forth by partisan news. As such, examining sociopolitical implications of news media is crucial [14], especially in times of heightened political divide in the United States and much of the western world.

According to diffusion theory, there are five stages by which people eventually come to adopt ideas [15,16]. This process begins with awareness, or knowledge, of an idea, primarily obtained through mass media. Arguably then, news media is a primary force in shaping knowledge about sociopolitical issues, and organizations can differ dramatically in how this “knowledge” is conveyed [17; for a real-world example see 18 compared to 19]. Content analyses show that right-leaning organizations (e.g., Fox News, Rush Limbaugh, Wall Street Journal) tend to use more “outrage language”, including insults, misrepresentative exaggeration, and character assassination [8,20], and framed immigrants more negatively after 9/11 [21], relative to left-leaning organizations (e.g., MSNBC, NPR, New York Times). Further, whereas content analyses largely focus on right-slanted news, such as Fox News [8,22–24], some find equally intense left-leaning slants and preferential treatment of Democratic presidential candidates on left-leaning outlets [24,25].

From an information processing perspective, partisan news is considered more persuasive than news providing multiple or balanced perspectives [26,27], in part because one-sided arguments (*vs*. information that includes counterarguments) are more likely to be accepted uncritically [28]. As such, partisan news exposure may result in congruent sociopolitical stances among consumers. For instance, the introduction of Fox News to a specific region coincided with increases in Republican candidate support in that area [14]. Right-wing news consumption is associated with more anti-immigrant attitudes [29] and climate change denial [30; see also ref. 31]. Moreover, partisan news exposure predicts views toward healthcare consistent with the news slant, regardless of the participants’ pre-existing ideology [26], increases opposition toward political candidates that disparaged by the outlet [24], and reinforces preferences for previously supported political candidate [32]. Moreover, there is debate as to whether left- and right-leaning news media exert an equivalent or a differing level of effect on consumers. Feldman and colleagues [31], for instance, found that that Fox News consumption is associated with greater climate change doubt and consumption of CNN and MSNBC is associated with greater acceptance of climate change, suggesting that right- and left-leaning media have similar degrees of influence on consumers. Moreover, Morris [33] found that audiences of both Fox News and CNN became increasingly polarized between 1998 and 2004. However, Hoewe and colleagues [34] found a stronger influence of right-leaning (vs. left-leaning) media, with Fox News consumption predicting endorsement of stricter anti-immigration policies, whereas neither MSNBC nor CNN exerted an effect on consumers’ views toward immigration.

Further, other work suggests some unique effects of media exposure. For instance, although *disparaging* news coverage of a political candidate may lead to more negative attitudes toward the candidate, it is less clear whether *favorable* coverage of a political candidate increases positive attitudes [24]. Others suggest that exposure to positive coverage of viewers’ non-preferred candidate may even backfire, leading to greater endorsement for their *prior* candidate preference [28,35,36]. Still others find no effect of Fox News on political attitudes among Republicans, Democrats, or Independents [37,38] or no effect of partisan newspaper consumption on voting [39].

Of note, most partisan news research has relied on cross-sectional correlational data drawing on convenience samples or on quasi-experimental work that relies on pre-existing groups (e.g., viewers of a particular broadcast). Further, most partisan media research relies exclusively on political candidate preference or voting patterns, which do not capture the complexity of polarization in the sociopolitical landscape (e.g., gun control, women’s rights, military support, immigration). Psychological research on media effects have also been criticized for frequent reliance on “ecologically invalid research materials, such as artificial laboratory-produced stimuli, rather than actual media” [40 (p.376); see also 41]. Indeed, experimental manipulations often rely on artificial text-based news articles that can be far-removed from the actual news content that people experience in the real-world [e.g., 42]. Other limitations include asking participants to select a single source from a limited range of options (e.g., do you watch Fox News or MSNBC?), and none, to our knowledge, has attempted to quantify the variety of news outlets consumed or length of exposure. As the diversity of news sources grows, such narrow focus on one or a few sources is unlikely to capture an ecologically valid reflection of partisan news use. Moreover, several attempts have been made to examine partisan news effects on political positions over time [e.g., 9,32] but lack statistical control for prior political positions. Therefore, it remains largely unclear whether, or to what extent, partisan media causally impact viewers’ positions on many polarizing issues.

Further, news organizations may modify their content to adapt to increasingly polarized audiences [2,4–7,43], and people may simply select news media that fits pre-existing preferences rather than partisan news impacting viewers over time. Rooted in selective exposure theory, this position posits that people prefer communications that are consistent with their pre-existing opinions [44,45]. Thus, right-leaning news consumption may be associated with right-leaning attitudes, not necessarily because media shapes consumer views over time, but because people right-leaning views choose these media. Indeed, people perceive themselves as largely impervious to the influence of mass media [46,47], and people on both sides of the political spectrum selectively expose themselves to attitude-consistent information, even when it comes at a cost [9,48–52]. For instance, Arendt and colleagues found that both explicit and implicit attitudes toward news outlets predict later choice in news articles, even when headlines from those sources were nearly identical [53], and Knobloch-Westerwick and Meng [54] found evidence for selective exposure to news media, regardless of the topic or political issue presented.

Underpinning these media effects one may expect to find motivated reasoning or motivated social cognition. Jost and colleagues [55], for instance, argue that ideologies may differentially be served by epistemic motives (i.e., to know and understand the world), existential motives (i.e., to survive and thrive in the world), and so-called ideological motives (i.e., rationalizing the status quo, attaining group or personal dominance). As such, people may be more or less drawn to certain types or flavors of media to satisfy psychological needs, and outlets that satisfy such needs are likely to subsequently shape attitudes and opinions. As such, people can be motivated by goals for accuracy but also by “directional” needs, that is, to reach particular conclusions. In the words of Kunda [56], “people are more likely to arrive at conclusions that they want to arrive at, but their ability to do so is constrained by their ability to construct seemingly reasonable justifications for these conclusions” (p. 480). From a motivated cognition viewpoint, media thus has the potential to draw in audiences seeking to reach particular conclusions, but also to provide the rationale for holding particular conclusions or forming opinions/attitudes. Thus, although partisan news may widen political gaps on polarizing issues, consistent with diffusion theory and information processing or motivated social cognition accounts, methodological limitations and the plausibility of selective exposure restrict our understanding of the causal direction between partisan news and viewers’ attitudes. As such, high quality data and rigorous methodologies are needed to determine the impact of partisan news on consumers.

### Present investigation

Here we examine relations between partisan news use, ideology, and attitudes, asking whether partisan news exposure can shape sociopolitical positions. We take a multi-method approach to address limitations in previous work and present results from cross-sectional (nationally representative), longitudinal, and experimental designs. In Study 1 we use nationally representative U.S. data to capture accurate and generalizable estimates of relations. In Study 2 we employ a 3-wave longitudinal method to test effects as they play out across time, isolating the unique effects of political news bias on future sociopolitical positions above pre-existing attitudes or ideologies. We also assess whether pre-existing attitudes predict future news use (i.e., a bidirectional model), consistent with selective exposure theory. Using an experiment in a supplemental study (S2 File), we assess whether exposure to real-world partisan news causally impacts sociopolitical positions, and whether this depends on viewers’ pre-existing political ideology. By experimentally manipulating exposure to real world news, this study addresses limitations of determining causality in past research and reliance on fabricated news content.

## Nationally representative cross-sectional associations (Study 1)

We predicted that more right-leaning news consumption would be associated with more right-leaning sociopolitical stances, and more left-leaning news consumption would be associated with more left-leaning sociopolitical stances. Attitudes toward immigrants, refugee admittance, the U.S. military, Muslims, feminists, gun control, and general political ideology were explored.

### Method

#### Participants and procedure

Nationally representative data from Americans (*N* = 4249) were provided by the 2016 American National Election Studies (ANES) Time Series Study (*M*_*age*_ = 49.31, *SD* = 17.33; 52.3% female; 71.1% White, 9.3% Black, 3.5% Asian/native Hawaiian/Pacific Islander, 10.5% Hispanic, 4.7% other race/multiple races). Participants indicated news outlet use and sociopolitical attitudes. Data can be obtained from the ANES website (https://electionstudies.org/data-center/2016-time-series-study/).

#### Partisan news use

Across studies, news source political leanings were determined by a Pew Research Center report [17]. ANES assessed consumption of the following right-leaning news organizations: Fox News, Sean Hannity Show, Rush Limbaugh Show, and Glenn Beck Program, and the following left-leaning news organizations: New York Times, Huffington Post, Washington Post, CNN, NBC, MSNBC, PBS, BBC, and Buzzfeed. This classification is consistent with previous classifications (e.g., refs. 8, 20, 21, 22, 23, 24, 25, 29, 30). Participants indicated which news sources they use at least once a month (1 = use, 0 = do not use), with responses summed to create indices of right-leaning and left-leaning news use.

Lean of news outlets can also be determined by AllSides (www.allsides.com), an organization that tracks bias in news media and incorporates editorial review, survey research, third-party data, and community feedback to classify American news sources as left-leaning, right-leaning, or centrist. These classifications are largely consistent with classifications made in the current report based on nationally representative data from Pew Research, with a few exceptions. Specifically, whereas Pew Research suggests that BBC and NPR are left-leaning, AllSides considers these sources as centrist. As such, analyses from Study 1 and Study 2 were re-run excluding these sources. Results suggest that removing these sources did not influence the results in any meaningful way, and as such we report findings using classifications based on Pew Research.

#### Political ideology

Assessing political ideology, participants classified themselves as extremely liberal to extremely conservative, and rated strength of identification as Democrat or Republican (both 7-point scales). These two correlated items (r = .70) were averaged into a single measure, with higher scores reflecting greater conservatism.

#### Anti-immigrant attitudes

Participants responded to the following: “Immigrants are generally good for America’s economy”, “America’s culture is generally harmed by immigrants”, and “Immigrants increase crime rates in the United States” (1 = agree strongly, 5 = disagree strongly). Responses were reversed and averaged, with higher scores reflecting more anti-immigrant attitudes (α = .79).

#### Anti-refugee attitudes

Participants indicated the extent to which they favor or oppose allowing Syrian refugees to come to the U.S. (1 = favor a great deal, 7 = oppose a great deal).

#### Military support

Participants indicated support for the U.S. sending ground troops to fight Islamic militants, such as ISIS, in Iraq and Syria (1 = oppose a great deal, 7 = favor a great deal).

#### Anti-muslim attitudes

Participants indicated how warmly (vs. coldly) they feel toward Muslims using a feeling thermometer (0–100). Scores were reversed, with higher scores reflecting more anti-Muslim attitudes.

#### Anti-feminist attitudes

Participants indicated how warmly (vs. coldly) they feel toward feminists using a feeling thermometer (0–100). Scores were reversed, with higher scores reflecting more anti-feminist attitudes.

#### Permissive gun attitudes

Participants indicated whether the government should make it easier to obtain a gun (coded as 3), keep the rules the same as they are now (coded as 2), or more difficult to obtain a gun (coded as 1). Higher scores indicate more permissive gun control attitudes.

#### Statistical approach

Examination of model variables revealed 15 outliers (0.4% of all data points), or values greater than three SD from the mean, on left-leaning news use, and 136 outliers (3.2% of all data points) on right-leaning news use. Outliers were winsorized (converted to the value at 3 SD from the mean). Missing data (0.4–16.2% for each variable) were estimated using FIML in Mplus v7.4 [57]. Regression analyses were conducted in Mplus v.7.4 using robust maximum likelihood estimation. Left-leaning news use and right-leaning news use were entered as simultaneous predictors of sociopolitical attitudes. Assumptions (normality, homoscedasticity, linearity, multicollinearity) were assessed via visual inspection of plots and statistical values (e.g., skewness, kurtosis, VIF) and were determined to be met for analyses in the current study and the following studies. Threshold for significance for all analyses for all studies was set at *p* = .05. Mplus code used to conduct this analysis and analyses in the following studies can be found in the S4 File.

### Results

See Table 1 for bivariate correlations. Regression results (Table 2) suggest that for all attitudes assessed, greater right-leaning news consumption was associated with more right-leaning stances and greater left-leaning news consumption was associated with more left-leaning stances. Of note, most associations between right-leaning news consumption and attitudes are as strong as those between left-leaning news consumption and attitudes, indicating some symmetry. However, right-leaning (*vs*. left-leaning) news consumption exhibited a stronger association with military support and conservatism, and left-leaning (*vs*. right-leaning) news consumption had a stronger association with lowered anti-immigrant and anti-Muslim prejudices. Overall, these findings demonstrate preliminary evidence that partisan news exposure may influence consumers’ views and contribute to political polarization. However, longitudinal and experimental investigations are needed to bolster this causal proposition.

**Table 1:** Correlations between variables (nationally representative cross-sectional data).

	1	2	3	4	5	6	7	8	9
1. Left-leaning news use									
2. Right-leaning news use	.13								
	[.10, .15]								
3. Conservatism	-.23	.38							
	[-.25, -.20]	[.37, .40]							
4. Anti-immigrant attitudes	-.34	.15	.37						
	[-.36, -.31]	[.13, .18]	[.35, .40]						
5. Anti-refugee attitudes	-.31	.28	.49	.56					
	[.34, -.29]	[.26, .30]	[.47, .51]	[.54, .58]					
6. Military support	-.11	.20	.30	.18	.18				
	[-.14, -.09]	[.18, .23]	[28, .33]	[.15, .21]	[.16, .21]				
7. Anti-Muslim attitudes	-.24	.16	.38	.48	.48	.14			
	[-.27, -.22]	[.14, .19]	[.35, .40]	[.45, .50]	[.46, .50}	[.11, .17]			
8. Anti-feminist attitudes	-.25	.25	.47	.36	.42	.19	.45		
	[-.28, -.23]	[.22, .27]	[.45, .49]	[.33, .38]	[.40, .44]	[.16, .21]	[.43, .48]		
9. Permissive gun attitudes	-.22	.24	.39	.30	.36	.15	.29	.36	
	[-.25, -.20]	[.21, .26]	[.37, .41]	[.28, .33]	[.34, .38]	[.12, .18]	[.26, .32]	[.33, .38]	
*M*	1.62	0.38	3.93	2.54	4.74	4.06	45.51	43.92	1.53
*SD*	1.78	0.68	1.82	0.98	2.04	2.04	25.37	26.01	0.62

*Note*. *N* = 4249. 95% confidence intervals are in parentheses. All *p*s < .001.

**Table 2:** Regression analysis predicting attitude positions (nationally representative cross-sectional data).

	β	SE	95% CI	p
**Anti-Immigrant**				
Right-leaning	0.20	0.02	[0.18, 0.22]	< .001
Left-leaning	-0.36	0.01	[-0.38, -0.34]	< .001
**Anti-Refugee**				
Right-leaning	0.33	0.01	[0.31, 0.35]	< .001
Left-leaning	-0.36	0.01	[-0.38, -0.33]	< .001
**Military Support**				
Right-leaning	0.22	0.02	[0.20, 0.24]	< .001
Left-leaning	-0.14	0.02	[-0.17, -0.12]	< .001
**Anti-Muslim**				
Right-leaning	0.20	0.02	[0.17, 0.22]	< .001
Left-leaning	-0.27	0.01	[-0.29, -0.25]	< .001
**Anti-Feminist**				
Right-leaning	0.28	0.02	[0.26, 0.31]	< .001
Left-leaning	-0.29	0.01	[-0.31, -0.27]	< .001
**Permissive Gun**				
Right-leaning	0.27	0.02	[0.25, 0.30]	< .001
Left-leaning	-0.26	0.01	[-0.28, -0.24]	< .001
**Conservatism**				
Right-leaning	0.39	0.01	[0.37, 0.41]	< .001
Left-leaning	-0.35	0.02	[-0.37, -0.32]	< .001

*Note*. Right-leaning = right-leaning news. Left-leaning = left-leaning news. Estimates are standardized.

## Cross-lagged longitudinal model (Study 2)

**Fig 1:**
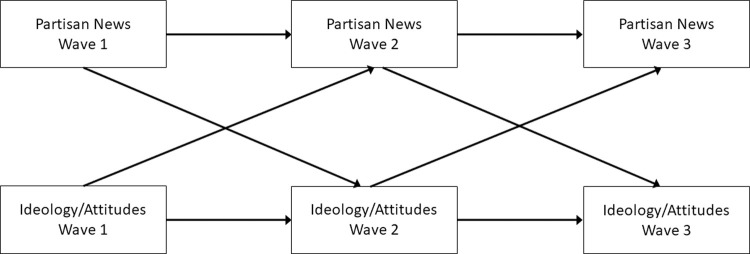
Conceptual figure showing auto-regressive and cross-lagged relations between news consumption, political ideology, and attitudes (longitudinal study). Correlations between variables (residuals for W2 and W3) within waves were modelled but not shown here for brevity.

Next we used a longitudinal approach predicting that, controlling for pre-existing sociopolitical stances, greater right-leaning news consumption would predict more right-leaning stances and greater left-leaning news consumption would be associated with more left-leaning stances, over time. Moreover, some scholars have suggested a “spiral” effect of news media, whereby news media both has the capacity to influence identity and attitudes, and identity and attitudes, in turn, influence choice in news media [58,59]. For instance, Westerwick and colleagues [60] found that people generally choose to view attitude-consistent information (selective exposure), and that exposure to attitude-consistent information reinforced these pre-existing attitudes. As such, we also assessed whether pre-existing sociopolitical positions predicts partisan news use over time (i.e., a bidirectional model; see Fig 1) to compare hypotheses regarding partisan news impacts (i.e., partisan news polarizes consumers) and selective exposure (i.e., polarized viewers select partisan news). We assessed attitudes toward Muslims, immigrants, terrorism, women’s rights, and gun control, all relevant to the news cycle during data collection.

## Method

### Participants and procedure

Data were collected from 650 American residents via Amazon Mechanical Turk (using Cloud Research) at three time points each spaced three months apart. To better ensure high quality data, Cloud Research includes verification of residency, the blocking of suspicious geocode locations, and the blocking of duplicate IP addresses. These checks were in place for the current study in addition to attention checks embedded within each survey to better ensure legitimate and reliable responses. Study 2 (and Study 3, see S2 File) sample sizes were pre-determined via power analysis conducted in Mplus v.7.4 [61] in which Monte Carlo simulations were used with effects estimated using results from Study 1, threshold significance set at p = .05, and power set at .80. In Study 2 and Study 3, ethics approval was obtained from the Brock University Research Ethics Board and written consent was obtained from each participant prior to participation in the study. At each wave, participants indicated news use and sociopolitical positions (see S3 File for full materials in Studies 2 & 3). Data can be obtained from https://osf.io/4k8s7/. Participants who failed attention checks at two or three waves were excluded (*n* = 16), as were participants self-categorizing as Muslim, immigrant, or a women’s rights activist (i.e., target groups), as per common practice in intergroup research (e.g., refs. [62,63]). After exclusions, the sample at Wave 1 (W1) comprised 484 participants (42.9% female, 57.1% male, M_*age*_ = 36.98, SD = 11.72; 80.6% White, 11.6% Black, 5.8% Asian, 5.8% Hispanic/Latino/South American, 1.6% another race/ethnicity; 55.2% Christian, 35.8% atheistic/agnostic, 6.6% practiced another religion). Of the full sample, 69.4% completed Wave 2 (W2), and 65.1% completed all three waves. Little’s test suggested that data were not missing completely at random (χ2 (680) = 762.49, *p* = .015). However, *t*-tests suggest that participants who completed all three waves did not differ from those who dropped out after W1 on any of the model variables (*p*s range from .093 to .993).

#### Partisan news use

Participants rated how often they used news sources over the last month (1 = *would never use*, 8 = *used daily*). New York Times, Huffington Post, Washington Post, NPR, CNN, NBC, MSNBC, PBS, BBC, Politico, Al Jazeera America, and New Yorker were averaged to create a measure of left-leaning news use (W1 α = .90, W2 α = .90, W3 α = .90). Use of Fox News, The Blaze, Rush Limbaugh Show, Glenn Beck Program, Drudge Report, and Breitbart were averaged to create a measure of right-leaning news use (W1 α = .83, W2 α = .84, W3 α = .83).

#### Political ideology

Participants indicated how liberal or conservative they are in social policy, economic policy, and in general, using 7-point Likert scales (ref. [64]; 1 = *very liberal*, 7 = *very conservative*; W1 α = .92, W2 α = .92, W3 α = .93).

#### Anti-muslim attitudes

The 7-item Modern Racism Scale [65] was adapted to tap anti-Muslim attitudes (e.g., “Muslims have more influence on government policies than they ought to have”; 1 = *strongly disagree*, 5 = *strongly agree*; W1 α = .92, W2 α = .93, W3 α = .93).

#### Anti-immigrant attitudes

Participants indicated anti-immigrant attitudes via a 5-item measure adapted from Pereira and colleagues [66] (e.g. “America is made a better place to live by people coming to live here from other countries”, reverse coded; 1 = *strongly disagree*, 7 = *strongly agree*; W1 α = .92, W2 α = .93, W3 α = .93).

#### Terrorism imminence beliefs

Beliefs in imminent terrorism were assessed using a 4-item measure adapted from Cox and Cheyne [67] (e.g., “I’m sure it’s only a matter of time before our country experiences another terrorist attack”; 1 = *strongly disagree*, 5 = *strongly agree*; W1 α = .73, W2 α = .79, W3 α = .73).

#### Anti-feminist attitudes

Negative attitudes towards women’s rights activists were assessed via a 5-item measure created for this study (e.g. “Women’s rights protesters are just complainers.” 1 = *strongly disagree*, 7 = *strongly agree*; W1 α = .92, *M* = 3.30, *SD* = 1.72; W2 α = .93, *M* = 3.41, *SD* = 1.78; W3 α = .94, *M* = 3.33, *SD* = 1.82).

#### Permissive gun attitudes

Permissive gun attitudes were assessed via a 5-item measure adapted from Igielnik and Brown [68] (e.g. “Being allowed to own a gun is essential to one’s sense of freedom:” 1 = strongly disagree, 7 = strongly agree; W1 α = .85, W2 α = .85, W3 α = .86).

#### Statistical approach

Outliers (27 values 3 SD from the mean) were winsorized. Missing data were estimated using FIML in Mplus v7.4. Using robust maximum likelihood estimation with Mplus we tested a cross-lagged panel model which included all lag-1 autoregressive paths (e.g., W1 political ideology predicts W2 political ideology, and W2 political ideology predicts W3 political ideology), all lag-1 cross-lagged paths (i.e., from news use to sociopolitical positions, and from sociopolitical positions to news use), and correlations between residuals within each wave. There was no significant decrement when constraining W1-W2 paths equal to W2-W3 paths for any given variable relative to when these paths were freely estimated (see S1 Table). We therefore retained the most parsimonious (i.e., constrained) model (for a similar procedure see refs. [69,70]).

### Results

Results for the crossed-lagged paths can be seen in Table 3, with autoregressive paths found in S1 Table. Controlling for pre-existing attitudes, greater right-leaning news consumption predicted more right-leaning stances on every issue assessed except terrorism imminence. Greater left-leaning news use predicted more left-leaning stances on all attitudes except terrorism imminence. Thus, in support of diffusion and information processing theories, the consumption of partisan news pulls consumers’ attitudes in the direction of the news slant over time. Moreover, associations for right- and left-leaning news are similarly strong, consistent with the general symmetry in Study 1. However, there are two notable exceptions; right-leaning (*vs*. left-leaning) news use was a particularly strong predictor of anti-women and anti-Muslim attitudes.

**Table 3:** W1-W2 and W2-W3 standardized cross-lagged paths in longitudinal model.

							95% CI	
			*β*	*SE*	*p*		Lower	Upper
Left-leaning news	→	Anti-immigrant	-0.05	0.02	.012		-0.09	-0.02
Left-leaning news	→	Pro-gun	-0.05	0.02	.004		-0.08	-0.02
Left-leaning news	→	Anti-women	-0.06	0.02	.004		-0.09	-0.02
Left-leaning news	→	Anti-Muslim	-0.07	0.02	< .001		-0.09	-0.04
Left-leaning news	→	Terrorism imminence	-0.03	0.03	.253		-0.08	0.01
Left-leaning news	→	Conservatism	-0.05	0.02	.003		-0.08	-0.02
Right-leaning news	→	Anti-immigrant	0.09	0.02	< .001		0.05	0.13
Right-leaning news	→	Pro-gun	0.07	0.02	< .001		0.04	0.10
Right-leaning news	→	Anti-women	0.08	0.02	< .001		0.05	0.12
Right-leaning news	→	Anti-Muslim	0.13	0.02	< .001		0.09	0.17
Right-leaning news	→	Terrorism imminence	0.04	0.03	.160		-0.01	0.08
Right-leaning news	→	Conservatism	0.09	0.02	< .001		0.05	0.13
Anti-immigrant	→	Left-leaning news	-0.03	0.03	.342		-0.08	0.02
Pro-gun	→	Left-leaning news	-0.06	0.03	.039		-0.10	-0.01
Anti-women	→	Left-leaning news	-0.05	0.03	.065		-0.09	-0.01
Anti-Muslim	→	Left-leaning news	0.03	0.03	.406		-0.03	0.08
Terrorism imminence	→	Left-leaning news	0.01	0.02	.523		-0.02	0.05
Conservatism	→	Left-leaning news	-0.04	0.03	.182		-0.09	0.01
Anti-immigrant	→	Right-leaning news	0.02	0.03	.637		-0.04	0.07
Pro-gun	→	Right-leaning news	0.02	0.02	.468		-0.02	0.06
Anti-women	→	Right-leaning news	0.00	0.02	.978		-0.04	0.04
Anti-Muslim	→	Right-leaning news	0.12	0.03	.001		0.06	0.17
Terrorism imminence	→	Right-leaning news	-0.02	0.02	.487		-0.05	0.02
Conservatism	→	Right-leaning news	0.05	0.03	.083		0.00	0.09

Effects control for autoregressive paths. Wave 1–2 (W1-W2) paths constrained to be equal to W2-W3 paths. (*N* = 484).

In contrast, there is less evidence that prior positions impacted choice in partisan news (after controlling for pre-existing news choice). More pro-gun attitudes predicted less use of left-leaning (but not right-leaning) news, and more anti-Muslim attitudes predicted greater consumption of right-leaning (but not left-leaning) news. However, no other attitude significantly predicted later partisan news consumption. Overall, rather than viewers selecting media suiting their pre-existing attitudes, partisan news appears to lead viewers to adopt perspectives increasingly consistent with the media they consume over time.

In the S2 File we provide details on an experiment among Canadian university undergraduates (largely White and female, approximately half identifying as Christian). We randomly assigned participants to view a 10-minute clip of actual media footage covering ISIS and Syrian refugees from either left-leaning or right-leaning news sources; those in the control viewed sports news. Only the right-leaning news coverage generated more anti-refugee attitudes, military support, and terrorism imminence beliefs (with no effects on Muslim attitudes). Data can be obtained from https://osf.io/4k8s7/.

## General discussion

As daily news can be markedly partisan and powerful in disseminating knowledge, thorough investigation of its effects is crucial, particularly against a backdrop of mixed findings. Our findings suggest that partisan news media can alter perceptions of reality, shape worries and sociopolitical positions, and widen political divides. First, large-scale cross-sectional data showed that greater consumption of right-leaning news was associated with greater conservatism and more right-leaning sociopolitical positions. In contrast, left-leaning news was associated with less conservatism and more left-leaning stances on politically polarizing issues. As nationally representative data, these findings offer high-quality point-estimates on the magnitude of these relations.

Next, using a longitudinal approach we found that, controlling for pre-existing sociopolitical positions, right-leaning news led to more negative attitudes toward immigrants, women’s rights, Muslims, gun control, and greater conservatism months later. Moreover, left-leaning news independently led people to adopt more positive attitudes toward immigrants, women’s rights, Muslims, gun control, and less conservatism months later. These findings suggest that political news leanings influence ideologies and attitudes of viewers, and that these effects are evidenced several months downstream. These effects (news → attitudes & ideology) were more reliable and robust than the reverse pathway. Partisan news appears able to shift one’s general political ideology to be more consistent with the political leaning of the outlet, which may result in greater polarization over time. In experimentally manipulating news exposure in the third study we found that right-leaning news (*vs*. control) caused increased terrorism imminence beliefs, anti-refugee attitudes, and support for military action against terrorism. The results from this Canadian experiment, however, warrant follow up with a nationally representative sample in the U.S., with equivalent numbers of men and women in the sample.

Overall, our findings demonstrate that associations between partisan news consumption and attitudes are not entirely due to selective exposure to belief-confirming information, but rather that partisan news sources can shift positions on politically polarizing issues in ways that can widen political divides on key sociopolitical issues. We observed some symmetries in the strength of associations between right-leaning news use and sociopolitical stance and between left-leaning news use and sociopolitical stances, but there were some notable and important exceptions. In particular, left-leaning (*vs*. right-leaning) news use had a particularly strong negative association with anti-immigrant and anti-Muslim attitudes, but only in Study 1. In contrast, right-leaning (*vs*. left-leaning) news use had a particularly strong association with some sociopolitical stances including military support and conservatism in Study 1, anti-Muslim attitudes in Study 2, and in Study 3 only the right-leaning (not left-leaning) news condition differed from the control. Thus, right-leaning (*vs*. left-leaning) news may have a particularly strong effect on certain sociopolitical attitudes. Future research on news media effects may explore the conditions under which left-leaning and right-leaning news consumption show symmetry or asymmetry in their impact on attitudes. Future research may also explore the effects of partisan media in other countries more culturally dissimilar to the United States or Canada, to assess cultural differences in news media effects or compare effects across different types of samples.

### Closing remarks

Deepening political polarization is cause for concern; solutions to contemporary problems such as the climate crisis and refugee resettlement require collaborative if not consensual views on the basic facts involved. Based on the present results we encourage news organizations to be mindful of their role in shaping social attitudes and beliefs about imminent harm. Consumers in turn must recognize differences between news outlets and appreciate how news shapes their reactions, something that does not come naturally [46,47]. If seeking to reduce political polarization, exposure to a variety or plurality of news outlets may be beneficial. Indeed, some theorists suggest that the effect of any single partisan news source may diminish when in the presence of other, competing news sources, and that such variety may balance to create an unbiased perspective [71–73]. With migration, gun rights, women’s rights, and political polarization representing pressing concerns in the 21^st^ century, reducing polarization may be critical in fostering global stability.

## Supporting information

S1 Fig Experimental effect of left-leaning news and right-leaning news exposure on social attitudes (Study 3). (PNG)

S1 Table W1-W2 and W2-W3 autoregressive paths from cross-lagged model. (DOCX)

S1 File Additional details regarding constraining paths (Study 2-longitudinal model). (DOCX)

S2 File Experimental manipulation (Study 3). (DOCX)

S3 File Study 2 and 3 materials. (DOCX)

S4 File Study 1, 2 and 3 Mplus code. (DOCX)
