# Supplementary figures and images for: News media impact on sociopolitical attitudes
Source: PLoS One. 2022 Mar 9;17(3):e0264031. doi: 10.1371/journal.pone.0264031 (PMC8906603; doi:10.1371/journal.pone.0264031)

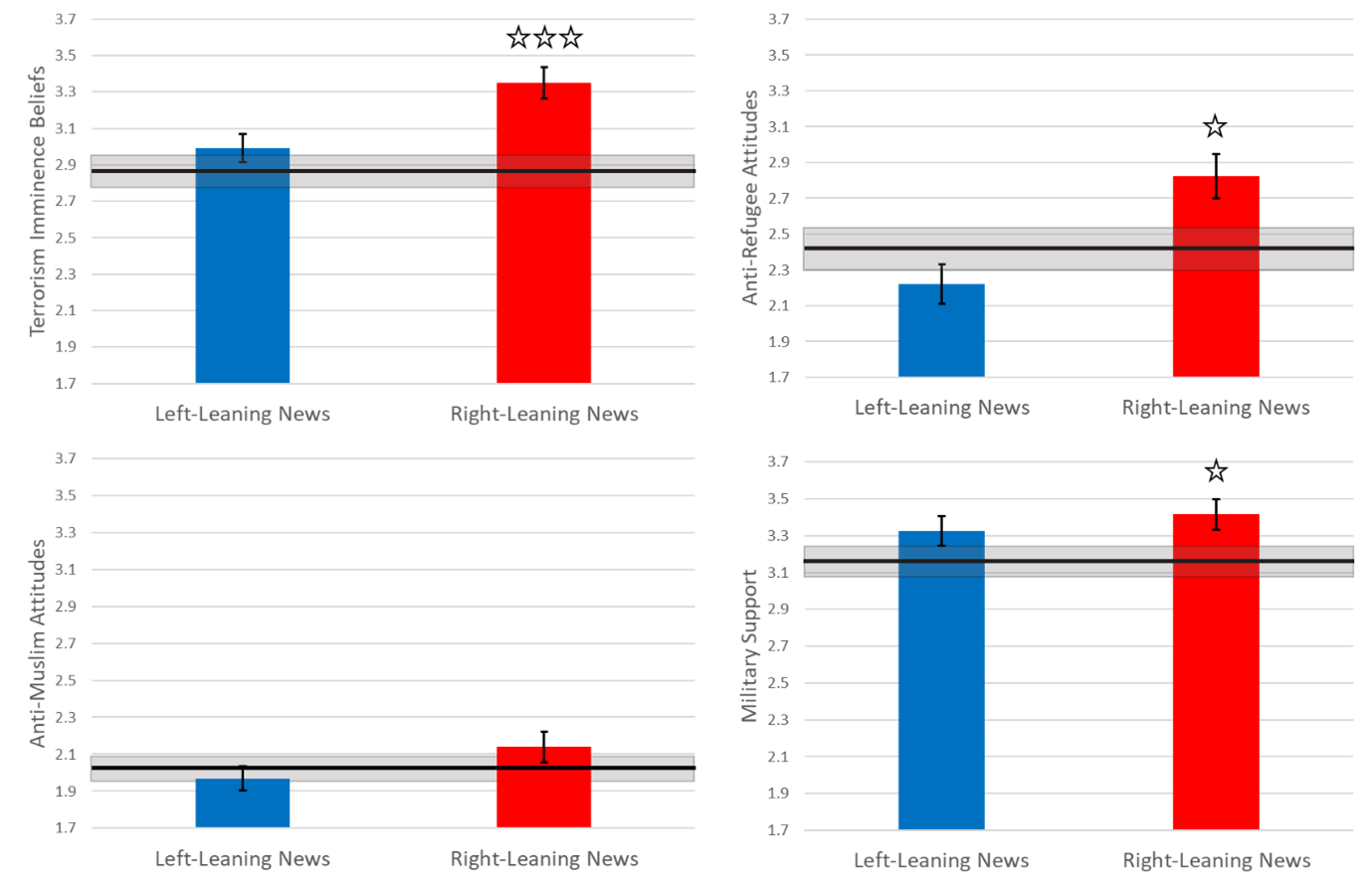

Supplement: S1 Fig — (PNG) [file pone.0264031.s001.png]
