# Supplementary material for: News media impact on sociopolitical attitudes
Source: PLoS One. 2022 Mar 9;17(3):e0264031. doi: 10.1371/journal.pone.0264031 (PMC8906603; doi:10.1371/journal.pone.0264031)
Supplement: S1 Table — (DOCX) [file pone.0264031.s002.docx]

**S1 Table. W1-W2 and W2-W3 autoregressive paths from cross-lagged model.**

|  |  |  |  |  | 95% CI | |
| --- | --- | --- | --- | --- | --- | --- |
|  | *β* | *SE* | *p* |  | Lower | Upper |
| Left-biased news | 0.77 | 0.03 | <.001 |  | 0.73 | 0.82 |
| Right-biased news | 0.75 | 0.03 | <.001 |  | 0.70 | 0.80 |
| Anti-immigrant | 0.80 | 0.02 | <.001 |  | 0.76 | 0.83 |
| Pro-gun | 0.87 | 0.02 | <.001 |  | 0.85 | 0.90 |
| Anti-women | 0.80 | 0.03 | <.001 |  | 0.76 | 0.85 |
| Anti-Muslim | 0.79 | 0.02 | <.001 |  | 0.75 | 0.83 |
| Terrorism imminence | 0.64 | 0.03 | <.001 |  | 0.58 | 0.69 |
| Conservatism | 0.87 | 0.02 | <.001 |  | 0.84 | 0.91 |

Cross-lagged paths can be seen in Table 2 of the main text. Wave 1-Wave 2 (W1-W2) paths constrained to be equal to the W2-W3 paths. (*N* = 484).
