# Supplementary material for: News media impact on sociopolitical attitudes
Source: PLoS One. 2022 Mar 9;17(3):e0264031. doi: 10.1371/journal.pone.0264031 (PMC8906603; doi:10.1371/journal.pone.0264031)
Supplement: S1 File — (DOCX) [file pone.0264031.s003.docx]

**S2 File. Additional details regarding constraining paths (Study 2- longitudinal model)**

There was no significant decrement when constraining W1-W2 autoregressive paths equal to W2-W3 paths for any given variable (χ2 (184) = 780.05, *p* < .001) relative to when these paths were freely estimated (χ2 (176) = 766.73, p < .001; Δ χ2 (8) = 12.89, *p* = .116). We therefore retained the most parsimonious (i.e., constrained) model (for similar procedure see Del Toro et al., 2019; Swart et al., 2011). We then added all 1-lag crossed paths to this autoregressive model (i.e., from news use to sociopolitical positions, and from sociopolitical positions to news use). Again, constraining the added paths to be equal across waves (χ2 (160) = 591.39, *p* < .001) did not significantly reduce model fit compared to the model in which these paths were freely estimated (χ2 (136) = 564.928, *p* < .001; Δ χ2 (24) = 30.94, *p* = .156), and we therefore report results for the most parsimonious (i.e., constrained) model.
