# Supplementary material for: News media impact on sociopolitical attitudes
Source: PLoS One. 2022 Mar 9;17(3):e0264031. doi: 10.1371/journal.pone.0264031 (PMC8906603; doi:10.1371/journal.pone.0264031)
Supplement: S2 File — (DOCX) [file pone.0264031.s004.docx]

**S3 File. Experimental manipulation (Study 3)**

We predicted that experimental exposure to right-leaning or left-leaning news would elicit more right-leaning or sociopolitical stances, respectively. Moreover, past work suggests that pre-existing political leanings can predict whether a consumer perceives bias in news media, and whether they perceive the content to be interesting and informative [1; see also ref. 2 for similar effects regarding attitudes toward vaccination]. As such ,we also assessed whether effects on attitudes depend on the prior-held political ideology of the viewer, specifically examining conservatism, right-wing authoritarianism (RWA: preference for tradition, convention, and authority; 3), and social dominance orientation (SDO; preference for social hierarchy and inequality; 4). In contrast to the reliance on fabricated news stimuli in past work, we utilize actual news content to understand the impact of partisan news in real life.

**Method**

**Participants and procedure**

Data were collected from 330 Canadian undergraduates. Participants were excluded for failing an attention check (*n* = 8) or technical error during testing (*n* = 3). Participants who self-categorized as Muslim, Syrian, or a refugee (target groups) were also excluded. Of the final sample (*n* = 305; M*_age_* = 20.27, SD = 4.16, 88.3% female), 71.8% were White, 8.1% were Black, 14.9% were Asian, 5.5% were Hispanic/Latino/South American, and 7.1% identified as another race/ethnicity. As for religious identification, 53.2% were Christian, 5.8% were Hindu, 31.8% were atheistic/agnostic, and 13.9% practiced another religion.

Participants first completed measures of political ideology, RWA, and SDO, then were randomly assigned to one of three conditions (left-leaning news *n* = 104; right-leaning news *n* = 96; or control condition *n* = 105) in which they watched three video clips. News conditions covered stories relevant at the time of data collection; namely terrorism and refugees from Syria. Participants then completed an attention check, followed by measures of anti-Muslim attitudes, anti-refugee attitudes, terrorism imminence beliefs, and military support, presented in random order. Participants then completed demographic information and a suspicion check.

**Political Ideology**

We assessed political conservatism as in Study 2 (α = .88). To assess RWA, a 12-item short-form measure (α = .86) adapted from Altemeyer [3] was used (e.g., “What our country really needs, instead of more ‘civil rights’ is a stiff dose of law and order”, 1 = *strongly disagree*, 7 = *strongly agree*). To assess SDO the 16-item measure (α = .87) [5] was used (e.g. “Some groups of people are simply inferior to other groups”, 1 = *strongly oppose*, 7 = *strongly favor*).

**Experimental Manipulation**

Video clips were taken from YouTube pages of real news outlets and, in some cases, shortened to approximately equivalent length but not modified in other ways. Clips from the left-leaning and right-leaning conditions were matched as closely as possible on objective details of the focal news stories and selected for representing popular news topics at the time of the study (ISIS, Syrian refugees). In the *left-leaning news condition* participants watched clips from Washington Post (2015; 1:37 minutes long), Huffington Post (2015; 1:50 minutes long), and CBC (2014; 2.50 minutes long; total duration 6:28). Participants in the *right-leaning news condition* watched news clips from Sun News Network, often referred to as “Fox News North” (Kinsella, 2012) (2015; 2:06 minutes long), Fox News (2016; 2:07 minutes long), and Global News (2014; 2:07 minutes long; total duration 6:33). In the *control condition* participants watched three clips of sports news commentary from the NBA (2016; 2:14 minutes long), the NFL (2016; 1:57 minutes), and the PGA (2016; 2:06 minutes; total duration 6:28). Pew Research suggests that Washington Post and Huffington Post are left-leaning and Fox News is right-leaning (Mitchell et al., 2014). In pilot testing, 23 experts in Canadian news sources anonymously rated the leaning of 33 outlets (1 = *very liberal*, 7 = *very conservative*), which revealed CBC as left-leaning (*M* = 3.00, *SD* = 1.24) and Sun News (*M* = 6.00, *SD* =1.05) and Global News (M = 4.14, SD = 1.15) as relatively more right-leaning.

**Anti-Muslim Attitudes**

Anti-Muslim attitudes were assessed as in Study 2 (α = .82).

**Anti-Refugee Attitudes**

Participants completed a 3-item measure of attitudes toward Syrian refugees (e.g., “Canadians will be put at risk if we allow more Syrian refugees into the country”; 1 = *strongly disagree*, 5 = *strongly agree*; α = .92).

**Terrorism Imminence Beliefs**

To assess terrorism imminence beliefs the measure from Study 2 was employed (α = .70).

**Military Support**

To assess support for military action against terrorism, an 8-item measure adapted from Kteily et al. [6] was used (e.g., “I support continued military efforts abroad to root out potential ISIS terrorists”; 1 = *strongly disagree*, 7 = *strongly agree*; α = .79).

**Suspicion Check**

Participants responded to four open-ended items including “Can you guess what the study was about?”. None guessed the hypotheses correctly.

**Statistical Approach**

Outliers (6 scores) were winsorized as in Studies 1-2. Missing data (0 - 4.9% for each variable) were estimated using FIML in Mplus v7.4. To test the hypothesized model, we used maximum likelihood estimation. Experimental condition was dummy coded (control = 0) to compare both the left-leaning and right-leaning conditions to the control condition. Models were fully-saturated (*df* = 0), and the outcome residuals were allowed to covary. Parameter estimates and significance tests were based on bias-corrected estimates generated from 1000 bootstrap samples.

**Results**

Participants exposed to right-leaning news (*vs*. control) reported more anti-refugee attitudes, military support, and terrorism imminence beliefs, but not anti-Muslim attitudes (see Fig 2 and Table 4). In contrast, the left-leaning news condition did not significantly differ from the control condition in terms of attitudes. We also conducted follow-up tests to determine whether effects depended on prior ideology (conservatism, RWA, SDO). The effect of news condition was not significantly moderated by participants’ previously held ideology (*p*s range .060 to .979). Results regarding the effects of experimental condition did not differ when any measure of ideology was included in the model. Thus, among both liberals and conservatives, exposure to right-leaning news on terrorism is enough to foster anti-refugee attitudes, beliefs that a terrorist attack is imminent, and greater military support.

**S4 Fig.** **Experimental effect of left-leaning news and right-leaning news exposure on social attitudes (Study 3).**

Error bars reflect standard errors. Mean and standard error for control condition represented by black line and shaded gray area respectively. Significance levels reflect contrasts with the control condition. (Study 3, *N* = 305, * *p* < .05, ** *p* < .01, ****p* < .001).

**S3 Table. Regression Analysis Predicting Attitude Positions (Experimental Study)**

|  |  | β | SE | 95% CI | p |
| --- | --- | --- | --- | --- | --- |
| **Terrorism Imminence** | |  |  |  |  |
| Right-leaning (*vs*. control) | | 0.27 | 0.06 | [0.18, 0.38] | <.001 |
| Left-leaning (*vs*. control) | | 0.07 | 0.06 | [-0.03, 0.17] | .215 |
|  |  |  |  |  |  |
| **Anti-Refugee** | |  |  |  |  |
| Right-leaning (*vs*. control) | | 0.16 | 0.06 | [0.05, 0.26] | .014 |
| Left-leaning (*vs*. control) | | -0.08 | 0.06 | [-0.19, 0.02] | .199 |
|  |  |  |  |  |  |
| **Anti-Muslim** | |  |  |  |  |
| Right-leaning (*vs*. control) | | 0.08 | 0.07 | [-0.14, 0.19] | .261 |
| Left-leaning (*vs*. control) | | -0.04 | 0.06 | [-0.15, 0.06] | .545 |
|  |  |  |  |  |  |
| **Military Support** | |  |  |  |  |
| Right-leaning (*vs*. control) | | 0.14 | 0.06 | [0.04, 0.24] | .023 |
| Left-leaning (*vs*. control) | | 0.10 | 0.07 | [-0.02, 0.20] | .161 |

Estimates are standardized.

**References**

1. Coe K, Tewksbury D, Bond BJ, Drogos KL, Prter RW, Yahn A, Zhang Y. Hostile news: Partisan use and perceptions of cable news programming. J Commun. 2008; 58: 201-219. doi: 10.1111/j.1460-2466.2008.00381.x
2. Gunther AC, Edgerly S, Akin H, Broesch JA. Partisan evaluation of partisan information. Comm Res. 2012; 39: 439-457. doi: 10.1177/0093650212441794
3. Altemeyer B. The authoritarian specter. Cambridge: Harvard University Press. 1996.
4. Sidanius J, Pratto F. Social Dominance: An Intergroup Theory of Social Hierarchy and Oppression. Cambridge: Cambridge University Press. 1999. doi: 10.1017/CBO9781139175043
5. Ho AK, Sidanius J, Kteily N, Sheehy-Skeffington J, Pratto F, Henkel KE ... Stewart AL. The nature of social dominance orientation: Theorizing and measuring preferences for intergroup inequality using the new SDO₇ scale. J. Pers. Soc. Psychol. 2015; 109: 1003-1028. doi: 10.1037/pspi0000033
6. Kteily N, Hodson G, Bruneau E. (2016). They see us as less than human: Metadehumanization predicts intergroup conflict via reciprocal dehumanization. J. Pers. Soc. Psychol*.* 2016; 110: 343-370. doi: 10.1037/pspa0000044
