# Supplementary material for: News media impact on sociopolitical attitudes
Source: PLoS One. 2022 Mar 9;17(3):e0264031. doi: 10.1371/journal.pone.0264031 (PMC8906603; doi:10.1371/journal.pone.0264031)
Supplement: S3 File — (DOCX) [file pone.0264031.s005.docx]

**S5 File. Study 2 and 3 materials.**

**Study 2 Materials**

Conservatism

1. Please indicate on the scale below how liberal or conservative (in terms of your general outlook) you are *in general*:

1                      2                      3                      4                      5                      6                      7 Prefer not to say

Very liberal                                                                                         Very conservative

2. How liberal or conservative do you tend to be when it comes to *social policy*

1                      2                      3                      4                      5                      6                      7 Prefer not to say

Very liberal                                                                                         Very conservative

3. How liberal or conservative do you tend to be when it comes to *economic policy*?

1                      2                      3                      4                      5                      6                      7 Prefer not to say

Very liberal                                                                                         Very conservative

4. Generally speaking, do you usually think of yourself as a Republican, Democrat, Centrist, or something else?

 Strongly Democrat

 Moderately Democrat

 Centrist (closer to Democrat)

 Centrist (neither Republican nor Democrat)

 Centrist (closer to Republican)

 Moderately Republican

 Strongly Republican

 Other (Please Specify) ___________________

 Decline to answer

News Use Measure

For each of the news sources, indicate which statement best reflects your usage of the source.

1= would never use

2 = would maybe use

3 = used once in the last month

4 = used 2-3 times in the last month

5 = used once a week, on average, in the last month

6 = used 2-4 times a week, on average, in the last month

7 = used 4-6 times a week, on average, in the last month

8 = used daily in the last month

9 = don’t know

10 = prefer not to say

1. Sean Hannity Show
2. CNN
3. FOX News
4. NBC
5. MSNBC
6. The Blaze
7. PBS
8. BBC
9. Rush Limbaugh Show
10. New York Times
11. Huffington Post
12. Glenn Beck Program
13. Washington Post
14. NPR
15. Politico
16. Drudge Report
17. Al Jazeera America
18. New Yorker
19. Breitbart

Attitudes toward Immigrants

1 2 3 4 5 6 7 prefer not to say

Strongly Moderately Slightly Neutral Slightly Moderately Strongly

Disagree Disagree Disagree Agree Agree Agree

1. It is good for America’s economy that people come to live here from other countries
2. America’s cultural life is generally enriched by people coming to live here from other countries
3. America is made a better place to live by people coming to live here from other countries
4. America’s crime problems are made worse by people coming to live here from other countries
5. People who come to live here generally take away jobs from workers in America

Modern Racism Scale

Please indicate your responses to the following questions.

1 2 3 4 5 Prefer not to say

Strongly Disagree Strongly Agree

1. Over the past few years, the government and news media have shown more respect for Muslims than they deserve.
2. It is easy to understand the anger of Muslims in America.
3. Discrimination against Muslims is no longer a problem in America.
4. Over the past few years, Muslims have gotten more economically than they deserve.
5. Muslims have more influence on government policies than they ought to have.
6. Muslims are getting too demanding in their push for equal rights.
7. Muslims should not push themselves where they are not wanted.

Terrorism Imminence Beliefs

Please indicate the extent to which you agree or disagree with the following statements.

1 2 3 4 5 Prefer not to say

Strongly Disagree Slightly Disagree Neutral Slightly Agree Strongly Agree

1. I’m sure it’s only a matter of time before our country experiences another terrorist attack.

2. The chances of the US being involved in a terrorist attack are quite large.

3. I am rarely worried about terrorist attacks in the US.

4. I am certain that the US is safe from terrorist attacks.

Attitudes toward Women’s Rights

1 2 3 4 5 6 7 prefer not to say

Strongly Moderately Slightly Neutral Slightly Moderately Strongly

Disagree Disagree Disagree Agree Agree Agree

1. The current women’s rights movements are completely justified.
2. Women’s rights activists are trying to gain superiority over men.
3. Women’s rights protesters are just complainers.
4. Women’s rights activists are making entirely reasonable demands.
5. Women’s rights protesters fail to appreciate the equality they already have in America.

Attitudes toward Guns

1 2 3 4 5 6 7 prefer not to say

Strongly Moderatley Slightly Neutral Slightly Moderately Strongly

Disagree Disagree Disagree Agree Agree Agree

1. It should be illegal for anyone to keep a handgun at home.
2. Being allowed to own a gun is essential to one’s sense of freedom.
3. “Concealed carry” should be allowed in more public places.
4. Teachers and officials should be allowed to carry guns in K-12 schools.
5. The ease with which people can legally buy guns contributes a great deal to gun violence in the country today.

**Study 3 Materials**

Conservatism

1. Please indicate on the scale below how liberal or conservative (in terms of your general outlook) you are *in general*:

1 2 3 4 5 6 7

Very liberal                                                                                  Very conservative

2. How liberal or conservative do you tend to be when it comes to *social policy*

   1 2 3 4 5 6 7

Very liberal                                                                                  Very conservative

3. How liberal or conservative do you tend to be when it comes to *economic policy*?

1 2 3 4 5 6 7

Very liberal                                                                                  Very conservative

Right-Wing Authoritarianism

Please circle your response, using the scale below.

| **0** | **1** | **2** | **3** | **4** | **5** | **6** | **7** |  |  |  |  |  |  |
| --- | --- | --- | --- | --- | --- | --- | --- | --- | --- | --- | --- | --- | --- |
| **Prefer not to say** | **Strongly**  **Disagree** | **Moderately**  **Disagree** | **Slightly**  **Disagree** | **Neither Disagree Nor Agree** | **Slightly**  **Agree** | **Moderately**  **Agree** | **Strongly**  **Agree** |  |  |  |  |  |  |

1. Gays and lesbians are just as healthy and moral as anybody else.

| **0** | **1** | **2** | **3** | **4** | **5** | **6** | **7** |
| --- | --- | --- | --- | --- | --- | --- | --- |

2. Atheists and others who have rebelled against the established religions are no doubt every bit as good and virtuous as those who attend church regularly.

| **0** | **1** | **2** | **3** | **4** | **5** | **6** | **7** |
| --- | --- | --- | --- | --- | --- | --- | --- |

3. There are many radical, immoral people in our country today who are trying to ruin it for their godless purposes, whom the authorities should put out of action.

| **0** | **1** | **2** | **3** | **4** | **5** | **6** | **7** |
| --- | --- | --- | --- | --- | --- | --- | --- |

4. Our country will be destroyed someday if we do not smash the perversions eating away at our

moral fibre and traditional beliefs.

| **0** | **1** | **2** | **3** | **4** | **5** | **6** | **7** |
| --- | --- | --- | --- | --- | --- | --- | --- |

5. The situation in our country is getting so serious, the strongest methods would be justified if they eliminated the troublemakers and got us back to our true path.

| **0** | **1** | **2** | **3** | **4** | **5** | **6** | **7** |
| --- | --- | --- | --- | --- | --- | --- | --- |

6. Everyone should have their own lifestyle, religious beliefs, and sexual preferences, even if it makes them different from everyone else.

| **0** | **1** | **2** | **3** | **4** | **5** | **6** | **7** |
| --- | --- | --- | --- | --- | --- | --- | --- |

7. People should pay less attention to the Bible and the other old traditional forms of religious guidance, and instead develop their own personal standards of what is moral and immoral.

| **0** | **1** | **2** | **3** | **4** | **5** | **6** | **7** |
| --- | --- | --- | --- | --- | --- | --- | --- |
|  |  |  |  |  |  |  |  |

8. The only way our country can get through the crisis ahead is to get back to our traditional values, put some tough leaders in power, and silence the troublemakers spreading bad ideas.

| **0** | **1** | **2** | **3** | **4** | **5** | **6** | **7** |
| --- | --- | --- | --- | --- | --- | --- | --- |

9. There is nothing wrong with premarital sexual intercourse.

| **0** | **1** | **2** | **3** | **4** | **5** | **6** | **7** |
| --- | --- | --- | --- | --- | --- | --- | --- |

10. What our country *really* needs, instead of more “civil rights” is a stiff dose of law and order.

| **0** | **1** | **2** | **3** | **4** | **5** | **6** | **7** |
| --- | --- | --- | --- | --- | --- | --- | --- |

11. Some of the best people in our country are those who are challenging our government, criticizing religion, and ignoring the “normal way” things are supposed to be done.

| **0** | **1** | **2** | **3** | **4** | **5** | **6** | **7** |
| --- | --- | --- | --- | --- | --- | --- | --- |

12. The facts on crime, sexual immorality, and the recent public disorders all show that we have to crack down harder on deviant groups and trouble-makers if we are going to save our moral standards and preserve law and order.

| **0** | **1** | **2** | **3** | **4** | **5** | **6** | **7** |
| --- | --- | --- | --- | --- | --- | --- | --- |

Social Dominance Orientation- 7 Scale

Show how much you favour or oppose each idea below by selecting a number from 1 to 7 on the scale below. You can work quickly; your first feeling is generally best.

1. Some groups of people must be kept in their place.

1 2 3 4 5 6 7

Strongly Somewhat Slightly Neutral Slightly Somewhat Strongly

Oppose Oppose Oppose Favour Favour Favour

2. It’s probably a good thing that certain groups are at the top and other groups are at the bottom.

1 2 3 4 5 6 7

Strongly Somewhat Slightly Neutral Slightly Somewhat Strongly

Oppose Oppose Oppose Favour Favour Favour

3. An ideal society requires some groups to be on top and others to be on the bottom.

1 2 3 4 5 6 7

Strongly Somewhat Slightly Neutral Slightly Somewhat Strongly

Oppose Oppose Oppose Favour Favour Favour

4. Some groups of people are simply inferior to other groups.

1 2 3 4 5 6 7

Strongly Somewhat Slightly Neutral Slightly Somewhat Strongly

Oppose Oppose Oppose Favour Favour Favour

5. Groups at the bottom are just as deserving as groups at the top.

1 2 3 4 5 6 7

Strongly Somewhat Slightly Neutral Slightly Somewhat Strongly

Oppose Oppose Oppose Favour Favour Favour

6. No one group should dominate in society.

1 2 3 4 5 6 7

Strongly Somewhat Slightly Neutral Slightly Somewhat Strongly

Oppose Oppose Oppose Favour Favour Favour

7. Groups at the bottom are just as deserving as groups at the top.

1 2 3 4 5 6 7

Strongly Somewhat Slightly Neutral Slightly Somewhat Strongly

Oppose Oppose Oppose Favour Favour Favour

8. Group dominance is a poor principle.

1 2 3 4 5 6 7

Strongly Somewhat Slightly Neutral Slightly Somewhat Strongly

Oppose Oppose Oppose Favour Favour Favour

9. We should not push for group equality.

1 2 3 4 5 6 7

Strongly Somewhat Slightly Neutral Slightly Somewhat Strongly

Oppose Oppose Oppose Favour Favour Favour

10. We shouldn’t try to guarantee that every group has the same quality of life.

1 2 3 4 5 6 7

Strongly Somewhat Slightly Neutral Slightly Somewhat Strongly

Oppose Oppose Oppose Favour Favour Favour

11. It is unjust to try to make groups equal.

1 2 3 4 5 6 7

Strongly Somewhat Slightly Neutral Slightly Somewhat Strongly

Oppose Oppose Oppose Favour Favour Favour

12. Group equality should not be our primary goal.

1 2 3 4 5 6 7

Strongly Somewhat Slightly Neutral Slightly Somewhat Strongly

Oppose Oppose Oppose Favour Favour Favour

13. We should work to give all groups an equal chance to succeed.

1 2 3 4 5 6 7

Strongly Somewhat Slightly Neutral Slightly Somewhat Strongly

Oppose Oppose Oppose Favour Favour Favour

14. We should do what we can to equalize conditions for different groups.

1 2 3 4 5 6 7

Strongly Somewhat Slightly Neutral Slightly Somewhat Strongly

Oppose Oppose Oppose Favour Favour Favour

15. No matter how much effort it takes, we ought to strive to ensure that all groups groups have the same chance in life.

1 2 3 4 5 6 7

Strongly Somewhat Slightly Neutral Slightly Somewhat Strongly

Oppose Oppose Oppose Favour Favour Favour

16. Group equality should be our ideal.

1 2 3 4 5 6 7

Strongly Somewhat Slightly Neutral Slightly Somewhat Strongly

Oppose Oppose Oppose Favour Favour Favour

Attention Check

What did the videos show?

1. Sports commentary
2. Terrorism and refugees
3. Anticipated changes in the economy
4. Celebrity gossip

IF CHOOSE A:

Which sports were shown?

1. Football, golf, soccer
2. Basketball, golf, swimming
3. Football, basketball, golf
4. Swimming, soccer, basketball

IF CHOOSE B:

Which topics were discussed?

1. Syrian refugees, financial support for ISIS, potential terrorism in Canada
2. Female ISIS fighters, journalist beheadings, Boston bombings
3. Financial support for ISIS, journalist beheadings, potential terrorism in Canada
4. Potential terrorism in Canada, female ISIS fighters, Boston bombings

Appendix E: Emotion

With regard to terrorism, to what extent do you feel:

Not at all Extremely

Angry 1 2 3 4 5 6 7

Scared 1 2 3 4 5 6 7

Outraged 1 2 3 4 5 6 7

Anxious 1 2 3 4 5 6 7

Furious 1 2 3 4 5 6 7

Afraid 1 2 3 4 5 6 7

:

Modern Racism Scale

Please indicate your responses to the following questions.

1. Over the past few years, the government and news media have shown more respect for Muslims than they deserve.

1 2 3 4 5

Strongly Disagree Strongly Agree

1. It is easy to understand the anger of Muslims in Canada.
   1. 2 3 4 5

Strongly Disagree Strongly Agree

1. Discrimination against Muslims is no longer a problem in Canada.

1 2 3 4 5

Strongly Disagree Strongly Agree

1. Over the past few years, Muslims have gotten more economically than they deserve.

1 2 3 4 5

Strongly Disagree Strongly Agree

1. Muslims have more influence on government policies than they ought to have.

1 2 3 4 5

Strongly Disagree Strongly Agree

1. Muslims are getting too demanding in their push for equal rights.

1 2 3 4 5

Strongly Disagree Strongly Agree

1. Muslims should not push themselves where they are not wanted.

1 2 3 4 5

Strongly Disagree Strongly Agree

Terrorism Imminence

Please indicate the extent to which you agree or disagree with the following statements.

1. I’m sure it’s only a matter of time before our country experiences another terrorist attack.

1 2 3 4 5

Strongly Disagree Slightly Disagree Neutral Slightly Agree Strongly Agree

2. The chances of Canada being involved in a terrorist attack are quite large.

1 2 3 4 5

Strongly Disagree Slightly Disagree Neutral Slightly Agree Strongly Agree

3. I am rarely worried about terrorist attacks in Canada.

1 2 3 4 5

Strongly Disagree Slightly Disagree Neutral Slightly Agree Strongly Agree

4. I am certain that Canada is safe from terrorist attacks.

1 2 3 4 5

Strongly Disagree Slightly Disagree Neutral Slightly Agree Strongly Agree

Military Support

Please indicate the extent to which you agree with the following items.

1. To put an end to terrorist acts by ISIS, I think it is okay to target civilians and fighters alike in foreign terrorist strongholds.

1 2 3 4 5 6 7

Strongly Strongly

Disagree Agree

1. To put an end to terrorist acts by ISIS, I think it is okay to bomb an entire country if it is known to harbor ISIS terrorists.

1 2 3 4 5 6 7

Strongly Strongly

Disagree Agree

1. We should spend more time on nonconfrontational efforts, as opposed to engaging in military activity, toward ISIS. (reverse-scored)

1 2 3 4 5 6 7

Strongly Strongly

Disagree Agree

1. I support continued military efforts abroad to seek out potential ISIS terrorists.

1 2 3 4 5 6 7

Strongly Strongly

Disagree Agree

1. We should not be afraid to hunt down any ISIS member who threatens any Western country anywhere.

1 2 3 4 5 6 7

Strongly Strongly

Disagree Agree

1. We are being too harsh towards ISIS. (reverse-scored)

1 2 3 4 5 6 7

Strongly Strongly

Disagree Agree

1. We should strike back with brutal force against members of ISIS who seek to intimidate us.

1 2 3 4 5 6 7

Strongly Strongly

Disagree Agree

1. There are ways to deal with ISIS without bringing in heavy artillery. (reverse-scored)

1 2 3 4 5 6 7

Strongly Strongly

Disagree Agree

Attitudes Toward Syrian Refugees

1. Allowing greater numbers of Syrian refugees in the country will pose a threat to Canada’s national security.

1 2 3 4 5

Strongly Disagree Slightly Disagree Neutral Slightly Agree Strongly Agree

1. Allowing greater numbers of Syrian refugees into Canada will NOT make our country any less safe.

1 2 3 4 5

Strongly Disagree Slightly Disagree Neutral Slightly Agree Strongly Agree

1. Canadians will be put at risk if we allow more Syrian refugees into the country.

1 2 3 4 5

Strongly Disagree Slightly Disagree Neutral Slightly Agree Strongly Agree
