# Supplementary material for: News media impact on sociopolitical attitudes
Source: PLoS One. 2022 Mar 9;17(3):e0264031. doi: 10.1371/journal.pone.0264031 (PMC8906603; doi:10.1371/journal.pone.0264031)
Supplement: S4 File — (DOCX) [file pone.0264031.s006.docx]

**S6 File. Study 1, 2 and 3 Mplus code.**

**Access information for Study 1**

Source: American National Election Studies (ANES)

Dataset name: 2016 Time Series Study

Link: <https://electionstudies.org/data-center/2016-time-series-study/>

**Relevant Variable Names:**

**Political Ideology**

- V16112
- 6V161158x

**News Outlets**

- v161370
- v161372
- v161409
- v161452
- v161430
- v161428
- v161433
- v161381
- v161403
- v161404
- v161415
- v161445
- v161379
- v161388
- v161390
- v161446
- v161386
- v161391
- v161393
- v161365
- v161429
- v161431
- v161432
- v161436
- v161405
- v161456
- v161451
- v161469
- v161482
- v161447
- v161454
- v161472
- v161485
- v161450

**Attitudes**

- V161214x
- V161213x
- V162096
- V162106
- V162268
- V162269
- V162270
- V161187

**Study 1 Mplus Code**

VARIABLE: NAMES ARE

libcon

party

libconparty

SR

MS

libnewsoutletwin

connewsoutletwin

femFTr

musFTr

imm

womenrightwin

permgun;

MISSING ARE all(999);

USEVARIABLES =

SR

MS

femFTr

musFTr

imm

permgun

libnewsoutletwin

connewsoutletwin

libcon;

ANALYSIS: ESTIMATOR = ML; Bootstrap = 1000;

MODEL:

imm on connewsoutletwin libnewsoutletwin;

SR on connewsoutletwin libnewsoutletwin;

MS on connewsoutletwin libnewsoutletwin;

musFTr on connewsoutletwin libnewsoutletwin;

femFTr on connewsoutletwin libnewsoutletwin;

permgun on connewsoutletwin libnewsoutletwin;

libcon on connewsoutletwin libnewsoutletwin;

libnewsoutletwin with connewsoutletwin;

OUTPUT: SAMPSTAT STANDARDIZED Cinterval;

**Study 2 Mplus Code**

VARIABLE: NAMES ARE

ID

polparty_w1

age_w1

gender_w1

ethnic_1_w1

ethnic_3_w1

ethnic_4_w1

ethnic_5_w1

ethnic_6_w1

ethnic_7_w1

ethnic_8_w1

ethnic_9_w1

rel_1_w1

rel_3_w1

rel_4_w1

rel_5_w1

rel_6_w1

rel_7_w1

rel_8_w1

rel_9_w1

immigrant_w1

womenact_w1

sexorient_w1

edu_w1

income_w1

polparty_w2

attcheck

libconw1

libconw2

libconw3

conneww1win

conneww2win

conneww3win

libneww1win

libneww2win

libneww3

immw1

immw2

immw3

gunw1

gunw2

gunw3

womw1

womw2

womw3

mrsw1

mrsw2

mrsw3

terrw1win

terrw2

terrw3;

MISSING ARE all (888 999);

!exclude Muslims, immigrants, women's rights activists

!and people who failed two or more attention checks

USEOBSERVATIONS =

rel_3_w1 ne 1 and immigrant_w1 ne 1 and womenact_w1 ne 1 and attcheck ne 0;

USEVARIABLES =

libconw1

libconw2

libconw3

conneww1win

conneww2win

conneww3win

libneww1win

libneww2win

libneww3

immw1

immw2

immw3

gunw1

gunw2

gunw3

womw1

womw2

womw3

mrsw1

mrsw2

mrsw3

terrw1win

terrw2

terrw3;

ANALYSIS: ESTIMATOR = MLR;

MODEL:

libconw2 on libconw1 (1);

libconw2 on libneww1win (2);

libconw2 on conneww1win (3);

conneww2win on conneww1win (4);

conneww2win on libconw1 (5);

conneww2win on immw1 (6);

conneww2win on gunw1 (7);

conneww2win on womw1 (8);

conneww2win on mrsw1 (9);

conneww2win on terrw1win (10);

libneww2win on libneww1win (11);

libneww2win on libconw1 (12);

libneww2win on immw1 (13);

libneww2win on gunw1 (14);

libneww2win on womw1 (15);

libneww2win on mrsw1 (16);

libneww2win on terrw1win (17);

immw2 on immw1 (18);

immw2 on libneww1win (19);

immw2 on conneww1win (20);

gunw2 on gunw1 (21);

gunw2 on libneww1win (22);

gunw2 on conneww1win (23);

womw2 on womw1 (24);

womw2 on libneww1win (25);

womw2 on conneww1win (26);

mrsw2 on mrsw1 (27);

mrsw2 on libneww1win (28);

mrsw2 on conneww1win (29);

terrw2 on terrw1win (30);

terrw2 on libneww1win (31);

terrw2 on conneww1win (32);

libconw3 on libconw2 (1);

libconw3 on libneww2win (2);

libconw3 on conneww2win (3);

conneww3win on conneww2win (4);

conneww3win on libconw2 (5);

conneww3win on immw2 (6);

conneww3win on gunw2 (7);

conneww3win on womw2 (8);

conneww3win on mrsw2 (9);

conneww3win on terrw2 (10);

libneww3 on libneww2win (11);

libneww3 on libconw2 (12);

libneww3 on immw2 (13);

libneww3 on gunw2 (14);

libneww3 on womw2 (15);

libneww3 on mrsw2 (16);

libneww3 on terrw2 (17);

immw3 on immw2 (18);

immw3 on libneww2win (19);

immw3 on conneww2win (20);

gunw3 on gunw2 (21);

gunw3 on libneww2win (22);

gunw3 on conneww2win (23);

womw3 on womw2 (24);

womw3 on libneww2win (25);

womw3 on conneww2win (26);

mrsw3 on mrsw2 (27);

mrsw3 on libneww2win (28);

mrsw3 on conneww2win (29);

terrw3 on terrw2 (30);

terrw3 on libneww2win (31);

terrw3 on conneww2win (32);

libconw1 with conneww1win libneww1win immw1 gunw1 womw1 mrsw1 terrw1win;

conneww1win with libneww1win immw1 gunw1 womw1 mrsw1 terrw1win;

libneww1win with immw1 gunw1 womw1 mrsw1 terrw1win;

immw1 with gunw1 womw1 mrsw1 terrw1win;

gunw1 with womw1 mrsw1 terrw1win;

womw1 with mrsw1 terrw1win;

mrsw1 with terrw1win;

libconw2 with conneww2win libneww2win immw2 gunw2 womw2 mrsw2 terrw2;

conneww2win with libneww2win immw2 gunw2 womw2 mrsw2 terrw2;

libneww2win with immw2 gunw2 womw2 mrsw2 terrw2;

immw2 with gunw2 womw2 mrsw2 terrw2;

gunw2 with womw2 mrsw2 terrw2;

womw2 with mrsw2 terrw2;

mrsw2 with terrw2;

libconw3 with conneww3win libneww3 immw3 gunw3 womw3 mrsw3 terrw3;

conneww3win with libneww3 immw3 gunw3 womw3 mrsw3 terrw3;

libneww3 with immw3 gunw3 womw3 mrsw3 terrw3;

immw3 with gunw3 womw3 mrsw3 terrw3;

gunw3 with womw3 mrsw3 terrw3;

womw3 with mrsw3 terrw3;

mrsw3 with terrw3;

OUTPUT: SAMPSTAT STANDARDIZED CINTERVAL;

**Study 3 Code**

VARIABLE: NAMES ARE

libcon

rwawin

sdowin

dc1

dc2

dc11

dc22

dc31

dc32

ef1

ef2

fear

anger

SR

MRSwin

MSwin

TIB

MT

IDinclude

MC1

followup

ethnic_7

refu

rel_2;

USEOBSERVATIONS =

idinclude eq 1 and

refu eq 2 and

rel_2 eq 0 and

ethnic_7 eq 0 and

MC1 LE 2 and

followup eq 1;

MISSING ARE all(999);

!dc1 dc2 = control as reference, dc11, dc22 = liberal as reference

! dc11 = control vs liberal, dc22 = conservative vs. liberal

! dc1= control vs liberal dc2 = control vs conservative

USEVARIABLES =

dc1

dc2

SR

MRSwin

MSwin

TIB;

ANALYSIS: ESTIMATOR = ML; Bootstrap = 1000;

MODEL:

!test if rwa moderates relation between condition and affect

!dc1 = lib vs. control, dc2 = con vs. control

SR on dc1;

MRSwin on dc1;

MSwin on dc1;

TIB on dc1;

SR on dc2;

MRSwin on dc2;

MSwin on dc2;

TIB on dc2;

OUTPUT: SAMPSTAT STANDARDIZED CINTERVAL(BCBootstrap);
